# Evaluating the relationship between neurological function, neuromuscular fatigue, and subjective performance measures in professional rugby union players

**DOI:** 10.3389/fspor.2022.1058326

**Published:** 2022-11-11

**Authors:** Ed Daly, Alan J. Pearce, Patrick Esser, Lisa Ryan

**Affiliations:** ^1^Department of Sport Exercise and Nutrition, Atlantic Technological University, Galway, Ireland; ^2^College of Sport, Health and Engineering, La Trobe University, Melbourne, VIC, Australia; ^3^Department of Sport, Health Sciences and Social Work, Centre for Movement, Occupation and Rehabilitation Sciences (MORES), Oxford Brookes University, Oxford, United Kingdom

**Keywords:** professional rugby players, fatigue, injury risk, readiness to train, RSI

## Abstract

Managing the health and wellbeing of full-time professional athletes is a multifaceted task. In elite high-performance environments, medical staff and strength training coaches attempt to identify improved methods to monitor player health. Monitoring player health could indicate potential injury risk and assist in adjustments to training and workload management. Measuring fatigue is a notable component of monitoring player readiness before and after training sessions, and after competitive fixtures. In the present study, a novel method of gathering non-invasive player data was investigated by utilizing the Omegawave (OW) to monitor direct current (DC) potential brainwave activity. This method allowed for non-invasive data gathering to assess recovery, player readiness and indicators of workload that may affect optimal performance. DC potential is based on recording low electrical frequencies (>0.5 Hz) that is derived from (1) Stabilization point of DC potential (mV), (2) Stabilization time (1.0–7.0) and (3) Curve shape (1.0–7.0). These measures evaluate the athlete's internal stress, readiness to perform, and neurological function through DC potential brain wave activity and heart rate variability (HRV) assessments. The primary aim of this case series was to compare the efficacy of objective DC potential brainwave activity measurements (neurological function) with neuromuscular fatigue data using reactive strength index modified (RSImod) and profile of mood states (POMS) questionnaires to assess a player's state of readiness to train. The participants in this study were eleven male senior professional rugby union players with a mean stature (±SD) of 185.2 ± 8.6 cm, mass of 101.1 ± 12.9 kg, and age of 27.1 ± 2.1 years. All players were tested 3 days per week over a 6-week mid-season period. Results from this case study suggest that DC potentials could be used as an objective measure to indicate player readiness and managing individual player workload. The final analyses identified a weak negative correlation (r = −0.17) between the RSImod data and the DC potential data was observed. DC potential brainwave activity data could be used in conjunction with subjective measures such as POMS, RSImod and reported injury status to adjust player daily activity.

## Introduction

Managing the health and wellbeing of full-time professional athletes is a time consuming and difficult task to achieve. In high performance, medical staff, sports scientists, and strength and conditioning coaches are continually seeking improved methods to monitor the health of their players and what injury risk they may be exposed to due to prior injury, training methods, time of the season and workload management. As rugby union has been a professional sport since 1995 the importance of player monitoring and tracking is paramount for clubs to remain competitive and retain a full roster of available players as the season evolves.

A significant component of monitoring player health and readiness is determining the workload tolerance of players are before and after training sessions, and competitive fixtures in league or championship (1). Sport scientists are constantly seeking new and more reliable methods to maintain players in an optimal manner. The monitoring of workload in players can offer coaches the means to optimize performance in training and in competitive periods during the season. This can manifest in various manners, for example being in a fatigued state will affect a player's ability to perform fundamental components of the game like tackling an opponent (2). Consequently, monitoring player workload around times where playing demands and injury risk is prevalent is a high priority in professional organizations (3). This cumulative workload will diminish the ability of the player to perform at an optimal level, result in a performance decrement and expose the players to injury risk.

In professional rugby organizations, multiple strategies are implemented to measure the overall functional state of readiness of players. Many professional sports teams will design preparatory programmes to elicit the required training load stresses for competing at an elite level. In general terms, these are divided into external loads applied and the internal stress measures as experienced by the players. The challenge to many practitioners is to gather and interpret these loads and stresses in a cogent manner that maintains player health and wellbeing.

When examining subjective or objective “readiness to train” markers, the practitioner is assessing an athlete's response to training. It is now well established that this is a multi-factorial approach, and it is highly recommended that multiple measures are considered to assess an athlete's “readiness to train” or reduce injury risk (4). A common measure to monitor external training load used by strength and conditioning practitioners are variants of the reactive strength index (RSI). RSI is an expression of lower body power and can be used a measure of fatigue related to external training load (5). RSI using drop jump or the reactive strength index modified using countermovement jumps are accepted methods that were primarily developed to demonstrate how athletes manage while performing various plyometric exercises (6). RSI using drop jumps are calculated as the ratio between jump height and ground contact time, while RSImod is calculated as a ratio between jump height and jump time. As mentioned, the monitoring of RSI variants can demonstrate improvement in athletic performance movement patterns and may be included as factor in determining an athletes' injury risk profile (7).

Self-report measures can be provided by the playing cohort in the form of profile of mood state (POMS) questionnaires. Concerns have been that self-reporting may not reflect accurate fatigue levels in players and more objective measures may provide more accurate data about the players (8). Further, compliance by players to completing POMS data may not be a sufficient measure of wellness in athletes and may be enhanced by using additional objective measures (9). Developing objective test protocols for measuring internal stress levels that players will experience in training or fixtures can be achieved using alternative invasive methods. Such as hormonal biomarkers to determine fatigue levels and/or state of readiness in conjunction that can be correlated with session data gathered by coaches (10). Many coaches and players will opt for non-invasive data gathering methods due to time, inconvenience and expense associated with biomarker data reference points. In this study, the method of gathering non-invasive player data was *via* Omegawave, Espoo, Finland (OW). This technology can monitor biopotential brainwave activity and is well established in the literature, dating to 1875, with the recording of bioelectrical activity in animal cerebral cortex studies (11, 12).

It has been suggested that the measurement of biopotentials (DC potential) could estimate the overall functional state of the central nervous system (CNS) (13). As this method is non-invasive approach that may detect variations in the biopotentials of cortical tissue. These variations in CNS states, for example fatigue, could detect possible alterations in an athlete's overall functional state (13). These measurements allow for non-invasive data gathering to assess recovery, overall player readiness and indicators of fatigue that may impede optimal performance. The OW technology uses proprietary algorithms that categorizes DC potential of brain biopotentials within a frequency range lower than the EEG range (0–0.5 Hz). The OW technology uses a proprietary algorithm which generates DC potential measures. These DC potential measures have been tested against similar technologies to establish sensitivity, validity and reliability [ICC (0.97) and significant correlation to EEG (r = 0.55; *p* < 0.001)] for the OW technology (14). These measures include DC potential curve shape (45 mV and higher), a DC stabilization point (1–3 min of initiating the test protocol) and DC stabilization time. The algorithm generates arbitrary units (AU) on a 1-7 scale for overall CNS readiness with higher values suggesting a higher level of activation of the CNS. Where the values are lower, this suggests that the athlete may not have recovered from previous training session stresses. These relatively uncomplicated scales (1-7) generate overall readiness measures which utilize DC potentials (curve, point, time) and heart rate variability (HRV). Previous research has utilized CNS readiness scores as an effective tool for monitoring athletic performance in elite athletes (5).

The primary aim of this case series in professional rugby was to compare the efficacy of objective DC potentials measurements, neuromuscular fatigue data (RSImod) and POMS questionnaires as a multifactorial means of monitoring player load and state of readiness. Combining DC potentials as a novel measurement approach (i.e., stabilization point and stabilization time) in tandem with external player load (RSImod) and POMS data could result in a multifactorial approach to monitor player readiness to train or perform.

## Materials and methods

### Participants

Eleven senior professional players with a mean stature (±SD) of 185.2 ± 8.6 cm, mass of 101.1 ± 12.9 kg, and age of 27.1 ± 2.1 years. Participant information sheets were distributed, and all participants provided voluntary informed consent prior to commencement of the study. The cohort of players were senior professional rugby players who were competing in the domestic league and European champions cup competition during the data collection period. In parallel with this, all players were involved with squad training sessions including strength & conditioning, recovery and unit skills sessions.

### Ethics and eligibility criteria

Ethical approval was received for this study *via* the Research Sub Committee of Atlantic Technological University (ATU_RSC_AC_201020_CR). Permission was granted by the Irish Rugby Football Union (IRFU) for the collection of data generated and for data supplied by the medical staff and coaches. Signed participant information forms were collected from each participant. It was established that all information would be treated as confidential and anonymised.

### Procedures

**Figure 1:**
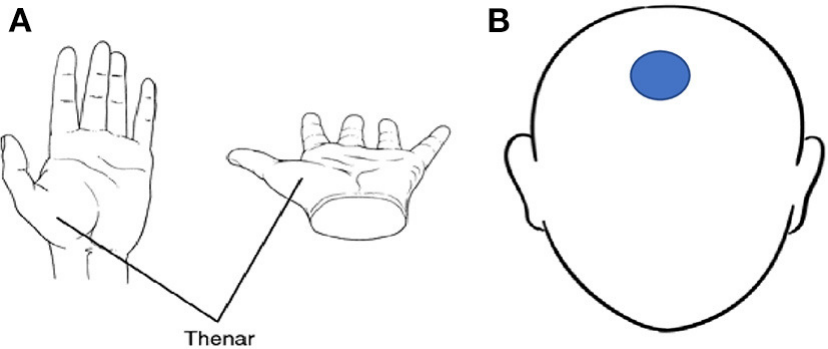
**(A)** Thenar eminence, **(B)** Electrode placement location on the forehead. Thenar image available from https://medical-dictionary.thefreedictionary.com/thenar.

For the purposes of this study, DC potential parameters (range from 0 to 0.5 Hz) were recorded using two recommended electrode placement sites. These sites were the forehead and the thenar eminence (radial side of the palm of the hand) of the participants. Consistency of electrode placement during the individual tests was assisted by separate and coded electrodes provided by the manufacturer indicating which electrode was to be placed on the head and the distal region of the thenar eminence (see Figure 1). Once the electrodes were in place, these cables were connected to the OW sensor on an electrocardiogram (ECG) strap that was placed around the chest of each participant (see section Data acquisition). The test area was a team room that was isolated from the remainder of the facility which provided a quiet environment and was maintained at a constant ambient temperature. Prior to the commencement of the data gathering, trial runs were completed to estimate the duration of the test sequence and troubleshoot issues with the test environment. Trial runs were completed by the primary researchers (*n* = 2) and members of the coaching staff (*n* = 2). RSImod data were gathered and monitored by the lead strength and conditioning coach for consistency of data capture. The RSImod data were collected using force decks (Vald, London, United Kingdom) that were *in situ* at the permanent training center for the professional squad. Each participant carried out three countermovement jump (CMJ) attempts per test day and an average RSImod were calculated from the three CMJs. Each participant self-reported their POMS data that were collated and recorded on a daily basis. These data were compiled on spreadsheets (Excel, MS Corporation, USA) per participant and monitored by members of the strength and conditioning team. Injury data were collected by the medical team with specific injuries (e.g., musculoskeletal injury) to participants classified according to the Orchard Sports Injury and Illness Classification System (OSIICS) (15). Illness (e.g., colds or flu) were self-reported to the medical team by the participants where a final illness diagnosis was recorded for ongoing monitoring purposes.

### Data acquisition

During the individual player test days on Monday, Tuesday, and Thursday of each week, (data were collected over a 6-week period) each player was given consistent instructions per each iteration of the test. Each player was to arrive in a fasted state and without caffeine or other stimulants and avoid stress prior to the test. Each data gathering session happened prior to squad training each morning which was generally between 7 and 8:30 am. The ECG chest strap electrode pads were immersed in water the strap was attached around the torso of each player aligning the strap with the midaxillary line of the body. An electrode was placed on the middle of the players forehead, and another single-use electrode was placed on the base of the thenar region of the right hand of each player (Figure 1). The head and hand electrodes (*via* cables) from the sensor on the ECG strap was paired with an Apple iPad using Bluetooth.

Players were requested to lie in a supine position on a physiotherapy plinth and were advised to breath as normal. The players were requested to stay in a supine position until the measurement was completed; this was indicated by a notification sound from the data collection device (iPad). The tester then selected the “save and analyse” option and the electrodes, along with the ECG strap was removed from each participant.

### Data analysis

Data were collated and the mean and standard deviations within individual player data and across the playing group (*n* = 11) throughout the testing period were calculated for multiple variables including CMJ, RSImod, POMS, DC potentials and injury rates. Data were compared between players on a daily and weekly basis and within and between players for the 6-week testing periods. Pearson's correlation coefficients were performed to assess associations between RSImod, DC potentials and POMS data.

## Results

The initial values derived from DC potential reflect the basic level of activation for the individual being assessed. The amplitude of the signal after stabilizes indicates the level of notional level of adaptability to stress and indicates the availability of energy reserves of the individual to perform activity (physical or cognitive). The data that were generated from the cohort were analyzed using Excel (MS Corporation) to investigate possible correlations between the RSImod data, self-reported POMS data and the objective DC potentials data.

Individual player data were correlated over the test period comparing RSImod data, DC potentials data and self-reported POMS data. Weekly averages of RSImod, DC potentials data and POMS data were correlated. The research team concluded that the most appropriate data would be comparisons of three metrics (RSImod, DC potentials data, and POMS data) over time and across all players that were tested during this case study.

DC potentials are based on brain energy that is derived from (1) Stabilization point (level/grade) of DC potential (mV), (2). DC Stabilization time (1.0-7.0) 3) Curve shape (1.0-7.0). For the purposes of the case series, the primary focus was on the values of the DC potential stabilization point as the primary measure.

The summarized data (see Table 1) identified a weak negative correlation (r = −0.17) between the RSImod data and the DC potentials (stabilization point). A weak negative correlation was observed (r = - 0.02) between overall fatigue (POMS) and DC potentials (stabilization point) along with a weak positive (r = 0.20) when correlating POMS and RSImod. Over the test period, DC potentials data generated greater standard deviation (±1.96) over the 6-week timeframe when compared to the RSImod (±0.02) and POMS (±0.44).

**Table 1:** All players (n = 11) summarised self-reported (POMS), DC potentials and reactive strength index modified (RSImod) data.

**Player**	**Position B/F**	**Height (m)**	**Mass (kg)**	**POMS** **(±SD) Fatigue** **(1-10)**	**POMS (±SD) Sleep Quality (1-10)**	**POMS** **(±SD)** **Overall Stress** **(1-10)**	**IN/IL (Y/N)**	**Mean DC** **(±SD)** **Time** **(minutes)**	**Mean (±SD) DC Potential (1-7)**	**Mean** **(±SD)** **FC:CT**	**Mean (±SD) RSImod**
1	B	1.87	98.4	1	7.50 (±0.83)	0.83 (±0.40)	N	2.12 (±0.47)	5.29 (±1.57)	0.80 (±0.02)	0.61 (±0.01)
2	B	1.84	90.1	1	8.85 (±0.37)	1.00	Y (IN)	2.28 (±0.49)	3.77 (±1.75)	0.80 (±0.02)	0.59 (±0.02)
3	B	1.83	87.3	2.35 (±0.63)	5.78 (±1.25)	2.42 (±0.51)	Y (IN)	2.10 (±1.29)	4.89 (±1.90)	0.81 (±0.03)	0.65 (±0.05)
4	F	1.78	102.4	1.66 (±0.52)	7.66 (±1.03	1.50 (±0.54)	Y (IN x 2)	2.12 (±0.50)	5.65 (±1.82)	0.82 (±0.04)	0.47 (±0.03)
5	B	1.7	74	2.14 (±0.69)	6.14 (±0.69)	3.57 (±0.53)	Y (ILX2))	1.75 (±0.70)	3.78 (±2.31)	0.74 (±0.07)	0.64 (±0.04)
6	F	1.83	117	1.75 (±0.50)	7.25 (±0.50)	1	Y(IN)	2.39 (±0.98)	5.28 (±1.96)	-	-
7	F	2.04	115.7	2.16 (±0.75)	5.16 (±1.47)	2.50 (±1.04)	Y (IN x 2)	2.64 (±1.15)	4.4 (±2.65)	0.83 (±0.03)	0.61 (±0.04)
8	F	1.83	117	2	7	1.50 (±0.54)	Y (IN x 2)	1.90 (±1)	2.87 (±1.94)	-	-
9	F	1.93	103	1.50 (±0.55)	6.50 (±2.58)	4.66 (±2.06)	Y(IN)	1.92 (±0.27)	3.90 (±2.35)	0.76 (±0.02)	0.59 (±0.02)
10	F	1.88	103.4	1.83 (±0.75)	5.33 (±1.21)	1.33 (±0.51)	N	1.79 (±0.70)	5.54 (±2.03)	0.74 (±0.04)	0.58 (±0.03)
11	B	1.88	97.6	1.60 (±0.54)	6.80 (±1.31)	1	Y(IN)	2.21 (±0.44)	5.59 (±1.31)	0.75 (±0.05)	0.55 (±0.02)

^**^Position B/F (Playing position (back / forward), **IN/IL Y/N** (Recorded injury / illness Yes/No) **FC:CT** (flight time:contraction time)).

### Player overview

The DC potentials data reflect the time required for the stabilization point to occur and indicates a transition from the active state to relaxed state. In this respect, the DC stabilization point can be regarded as the general state of readiness of each player. The results from this cohort suggest there were great greater variance in DC potentials (neurological function) when compared to RSImod (neuromuscular function) or POMS (self-reported). The RSImod and POMs measures displayed a consistency in values across the participants over time. An example of this is Player 5 who recorded two bouts of illness. It is notable in relation to the consistency of the POMS data for fatigue, sleep quality and stress (±0.69, ±0.69, and ±0.53) and the RSImod data (±0.04) which were recorded. The DC stabilization point for Player 5 during the recorded test sessions displayed a greater variance (±2.31) over the same time period. Similar data were recorded for players 2, 3, and 11 with little variance in RSImod or POMS (overall fatigue, sleep quality or stress measures).Player 2 recorded similar scores for sleep quality (± 0.37) and RSImod (±0.02), while the DC stabilization point for player 2 was ±1.75. Player 3 recorded similar values for overall fatigue (±0.63), overall stress (±0.51) and RSImod (±0.05) and a DC stabilization point of ±1.90. The trend for little variance in POMS and RSImod data were recorded for player 11 with overall fatigue (±0.5) and an RSImod value of ±0.02. Player 11 recorded a DC stabilization point of ±1.31.

Player 9 recorded little variance in the RSImod (±0.02) over time. This player had similar values in sleep quality, stress and DC stabilization point (±2.5, ±2.06 and ±2.35 respectively). Player 1 recorded similar values for sleep quality (±0.83) and overall stress (±0.40) and a DC potential value of ±1.57. Player 10 recorded a similar value for overall fatigue (±0.75) and overall stress (±0.51) and a notable variance on DC stabilization point (±2.03).

Nine out of eleven players (81.8%) either reported an injury or illness or both injury and illness throughout the test period. Within these nine players, there were four players that reported either injury or illness on two occasions (Players 4, 5, 7, and 8). The injuries sustained by players 6 and 8 (lower limb) impeded these players from recording FC:CT and subsequently RSImod values. The POMS data for fatigue, sleep quality and stress for Player 6 (±0.50, ±0.50, 0, respectively) was not reflected in the DC stabilization point data (±1.96). Player 8 displayed a similar profile across the POMS data (0,0, ±0.54) compared to the DC stabilization point (±1.94).

### Case study: Player 4

Player 4 was diagnosed with injuries on week 4 of the test period (lower limb) and week 5 (concussion) of the test period. Over the test period, the self-reported measures of POMS for three categories (i.e., fatigue, sleep quality and overall stress) displayed minimal variation with standard deviations of 0.52, 1.03, and 0.54 respectively. The test of neuromuscular function (i.e., flight time:contraction time, RSImod) were also consistent for the same time period with standard deviation values of 0.04 and 0.03, respectively. DC potentials values across the test period were more variable with reported standard deviations of 1.04 (DC potential overall readiness) and 1.82 (DC stabilization point) during the same timeframe suggesting that the DC potentials may be more sensitive to fatigue than the other measures.

Observing the data during the week preceding the injury incidences (lower limb, week 3 and concussion in week 5). The POMS Fatigue were an average score of three (low levels of self-reported fatigue), sleep quality were an average of six (self-reported high quality). Player 4 recorded an average DC Stabilization Point of 6.76 and DC Stabilization Time (minutes) were an average of 169 s (moderate readiness). During week 3, they recorded an average RSImod of 0.49.

In week 5, Player 4s self-reported POMS Fatigue were an average of 2.5, the POMS Sleep were self-reported as 6. DC Stabilization Point averaged 6.1 and had an average DC Stabilization Time of 2.12 min (moderate state of readiness). Player 4 did not record RSImod during week 5 due to the head injury (concussion) sustained.

## Discussion

This case series in professional rugby union players attempted to evaluate the efficacy of objective DC potential measurements, neuromuscular fatigue data (RSImod) and self-reported data (POMS) as a comprehensive approach to monitoring player workload. DC potentials were the novel measurement approach in conjunction with external player load (RSImod) and self-reported POMS data. The DC potential metrics could provide coaches with parameters that suggest optimum states of readiness and when athletes have favorable adaptive capabilities (5). In this study, the DC potentials data provided additional objective data that could be compared with the existing readiness protocols. The DC potentials data appeared to be more sensitive to changes in the players states of readiness over the 6-week test period.

DC potential brainwave activity attempts to direct an athlete's readiness to perform, levels of internal stresses, cardiac measurements and offer a measurable insight into the neurological function of athletes (13). For the purposes of this study, longitudinal POMS data were gathered, however much of the POMs data were subjectively uniform suggesting that the POMS data would not provide a reliable marker for player monitoring purposes. In this case study, players tended to complete the POMS data as a matter of course without giving due diligence to the completion of the data. As many athletes in this study reported little deviation and recorded a very narrow range of values while with other players, there was a clear alteration in the POMS values that they self-reported. The management of players' readiness to train offers internal or external markers (subjective or objective markers). Subjective measures such as mood state, sleep disturbance or perceived levels of external stress are generally regarded as having a high degree of reliability with objective measures once suitably monitored (16). In this case study, the lack of variance across the cohort of players was notable, this may require additional scrutiny by coaching staff to ensure more accurate POMS values are reported. In this instance, it could be suggested that when POMS goes unmonitored, players gave little attention to inputting accurate values.

In dynamic high-performance environments such as professional rugby union franchises, it is important to individualize training load by using multiple tools to monitor individual workload responses (17). Where excessive fatigue goes unmonitored over protracted periods of time, this may lead exposure to injury risk including overreaching or overtraining which will impact on performance (18). When fatigue levels are not accurately monitored and tracked, this could lead to a decrement in overall performance and injury risk (19). When examining the values derived from the objective RSImod (CMJ-countermovement vertical jump) to determine the effect of workload and fatigue on neuromuscular performance. These data were highly consistent across most players for the duration of the test period. This was notable as many of the players (81.8%) recorded either an illness of an injury or both during the test period. In the majority of players' data, there was little variance observed in the RSImod scores over time.

The DC potentials data displayed variance for the majority of players that was not evident by the POMS or RSImod data points. Selective multiple markers should be recorded and monitored, particularly markers that are objective and cannot be manipulated by player efforts or inputs. It appears that the data derived from this case study promotes the use of more that objective markers, such as DC potentials. Where high performance is the central focus, it could be argued that utilizing DC potential measures may inform the more accepted and established self-report measures. In addition, the individual players' coefficient of variation (CV) could be used to assess if there is a reduction in the “readiness to train” parameters. Where meaningful changes are detected and observed in individual players, the practitioner could alter the volume & intensity of workload.

### Limitations and future research

As this was a case study, it is not clear whether we can categorically state that neurological function (DC potentials) could be used as a component in player monitoring. We can suggest that DC potentials are more sensitive to changes in professional rugby union players when compared to a neuromuscular measure such as RSImod or self-reported measures (POMS). A further limitation of the case study is the duration, all data was gathered over a 6-week period. This length of time was not sufficient to establish long term patterns in the cohort of players. In order to identify the possibility that DC potentials are a viable objective measure, a longitudinal data would be required over the course of multiple competitive seasons.

For long term studies, a larger cohort of players would be required with the inclusion of a control group or alternative fatiguing protocol. A final limitation of the study was a full understanding of the proprietary algorithm and how calculations were derived by the product. In conclusion, this study supports the position that the most comprehensive means to monitor individual player workload is a multi-modality approach. This multimodal approach would involve the use of self-reported measures (POMS), a measure that can detect alterations in neuromuscular function (RSI or RSImod), in addition to other sources of objective data that may improve player welfare. This case series suggests that the inclusion of DC potentials would be an option as an additional objective measure as they have demonstrated promise regarding an increased sensitivity to indicating athlete readiness. The findings from this case series suggest that OW may be used as adjunct tool to current established protocols. The results found in this case series argue for increased research using observational and suitably powered sample sizes.

## Data availability statement

The original contributions presented in the study are included in the article/supplementary material, further inquiries can be directed to the corresponding author.

## Ethics statement

The studies involving human participants were reviewed and approved by Research Subcommittee of Atlantic Technological University. The patients/participants provided their written informed consent to participate in this study.

## Author contributions

ED and LR contributed to concept and design of the study, data acquisition, data analysis, and the writing of the manuscript. AP and PE contributed to data analysis and the writing of the manuscript. ED, LR, AP, and PE reviewed and edited previous drafts of the manuscript. All authors read the final version of the article and approved the submitted version.

## Conflict of interest

Author AP currently receives partial research salary funding from ERASMUS+ Partnership Grant (2019-1-IE01-KA202-051555) and is a Director for NeuroSports Labs (Australia) and Concussion Legacy Foundation Australia. Author AP has previously received partial research funding from Sports Health Check charity (Australia), the Australian Football League, Impact Technologies Inc. (Australia), and Samsung Corporation, and has provided expert testimony to courts on concussion injury. The remaining authors declare that the research was conducted in the absence of any commercial or financial relationships that could be construed as a potential conflict of interest.

## Publisher's note

All claims expressed in this article are solely those of the authors and do not necessarily represent those of their affiliated organizations, or those of the publisher, the editors and the reviewers. Any product that may be evaluated in this article, or claim that may be made by its manufacturer, is not guaranteed or endorsed by the publisher.
